# 2253. Hospitalist Antibiotic Prescribing Patterns by Hour and Weekday

**DOI:** 10.1093/ofid/ofad500.1875

**Published:** 2023-11-27

**Authors:** Joshua H Gray, Lana Wahid, Rebekah W Moehring, Michael E Yarrington

**Affiliations:** Duke University Health System, Durham, North Carolina; Duke University School of Medicine, Durham, North Carolina; Duke University, Durham, NC; Duke University Health System, Durham, North Carolina

## Abstract

**Background:**

Hospital medicine physicians providing coverage during evening and night shifts are often responsible for higher patient volumes during these busy shifts. Data on decisions to start or broaden antibiotic therapy by covering physicians are limited.

**Methods:**

We evaluated antibiotic administration data from Duke University Health System between July 1, 2021 and March 31, 2023 to identify antibiotic “starts” and “escalations” attributed to hospitalists. Starts were defined as the first administration per patient encounter without antibiotics on the preceding calendar day. Escalations were defined as an increase in cumulative spectrum score (using a previously validated antibiotic spectrum index) in the 48 hours following administration compared to the score in the 48 hours prior. Shifts from intravenous to only oral antibiotics were not counted as an escalation. We visualized these data as a heat map based on weekday-hour combinations and then compared the median number of starts and escalations between daytime shifts (weekdays 7AM to 5PM, weekends 7AM to 2PM), evening, “cross cover,” coverage shifts (weekdays 5PM to 10PM, weekends 2PM to 10PM), and overnight shifts (remaining times), using the Mann-Whitney U nonparametric test with daytime shifts as the comparator group.

**Results:**

The analysis included 7,045 antibiotic starts and 4,879 escalations across 78,845 unique patient admissions. Heat maps (Figure 1A and B) revealed specific practice patterns. Median starts for night shift were significantly lower than day shift starts, but cross cover starts were significantly higher (p = 0.011 and p < 0.001, respectively, Figure 2A). Night shift escalations were significantly lower than day shift while cross coverage showed no difference (p< 0.001 and p = 0.51, respectively, Figure 2B).

Antibiotic starts and escalations by hour and day of week

**Figure:**
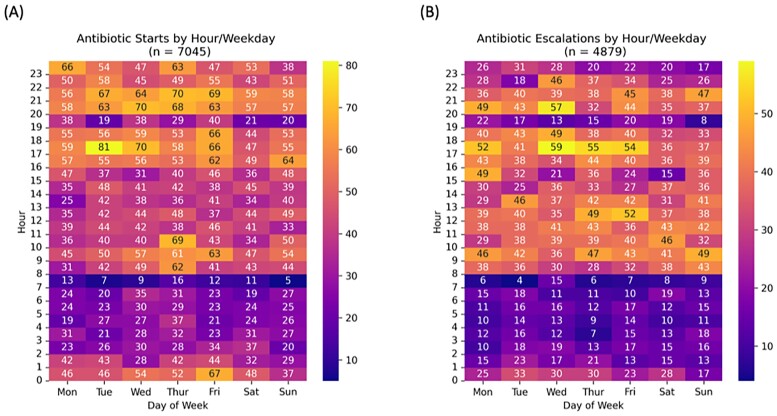

Heat maps of counts of antibiotic (A) starts and (B) escalations by hour and day of week

Hourly starts and escalations of antibiotics by hospitalist shift

**Figure:**
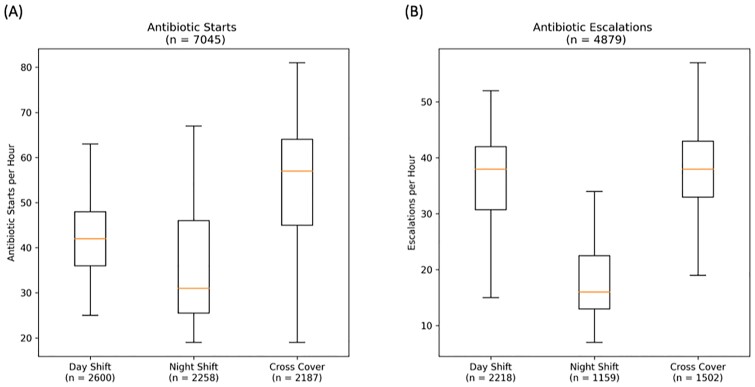

Box-and-whisker plots of counts of antibiotic (A) starts and (B) escalations per hour by hospitalist shift.

**Conclusion:**

Antibiotic escalations differed across work shifts; visualization of these patterns aids in antimicrobial prescribing pattern recognition and may assist in finding opportunities for supportive antimicrobial stewardship strategies.

**Disclosures:**

**Rebekah W. Moehring, MD, MPH, FIDSA, FSHEA**, UpToDate, Inc.: Author Royalties

